# Morgan David Enoch, MBBS, DPM, FRCPsych

**DOI:** 10.1192/bjb.2025.10168

**Published:** 2026-04

**Authors:** Judith R. Harrison

Formerly Emeritus Consultant Psychiatrist, Royal Liverpool University Hospital, UK

**Figure:**
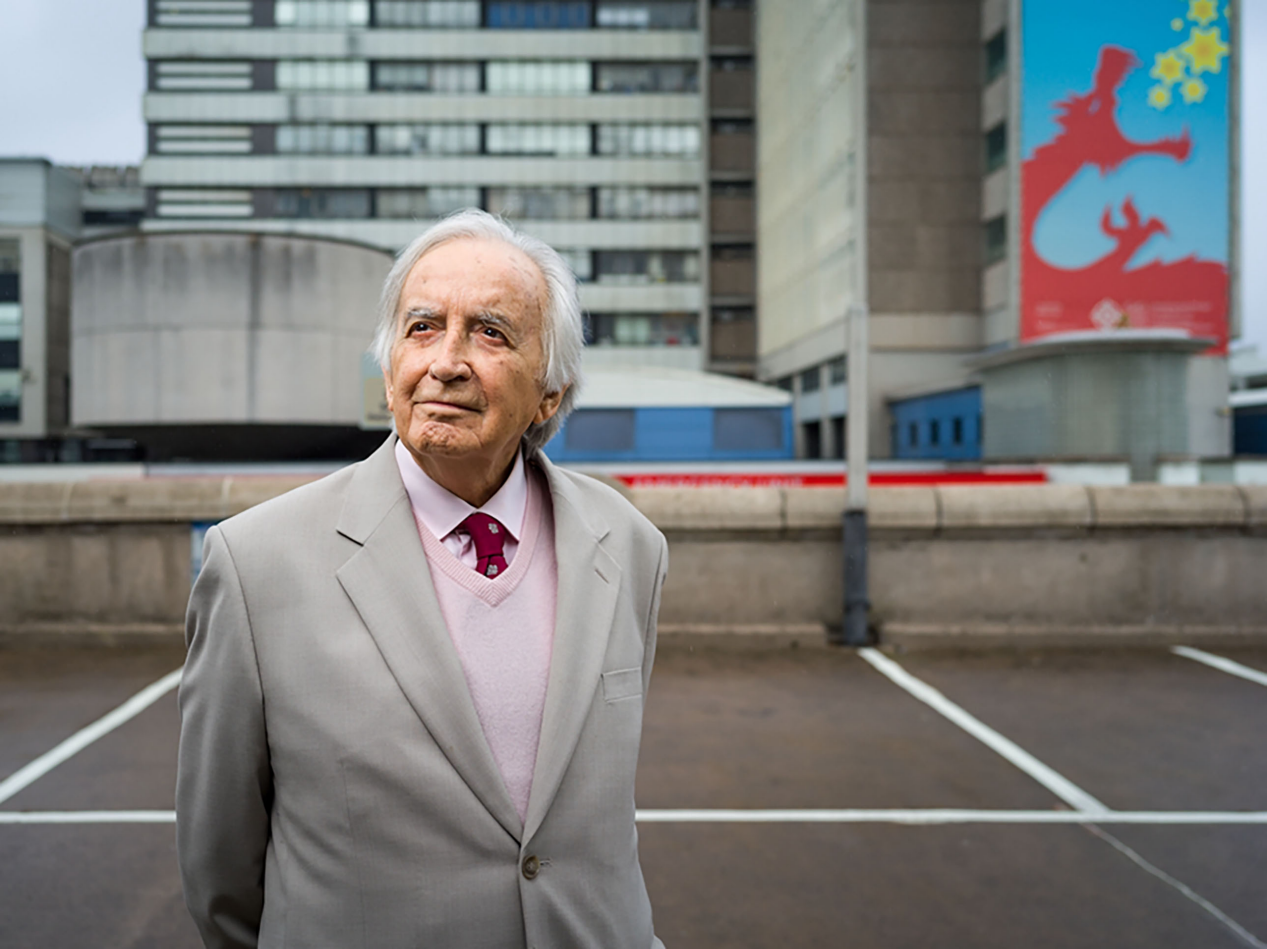
Dr M. David Enoch, at the University Hospital of Wales, 2018. Photographer: Benjamin Gilbert. Source: Wellcome Collection. https://wellcomecollection.org/stories/fees--funding-and-the-nhs. Attribution-NonCommercial 4.0 International (CC BY-NC 4.0).

Morgan David Enoch, usually called David, who has died aged 99, was a tenacious campaigner for humane mental healthcare. As a young consultant he exposed overcrowded wards and appalling conditions in long-stay hospitals, politely but persistently lobbying politicians such as Minister of Health Enoch Powell to close the asylums and invest in community services. His activism, wryly dismissed by ministers including Sir Kenneth Robinson as troublemaking, helped lay the groundwork for Britain’s shift towards care in the community.

Born in 1926 in Penygroes, South Wales, David was the son of Tom, a coal hewer, and his wife, Maisy. Tom was adamant that his son should not follow him down the pit, and David was a sixth-form student planning to read theology when, just before the end of the Second World War, a letter from the king conscripted him into the army. After training at the Indian Military Academy in Dehradun, he was commissioned into the Royal Artillery and posted to the Second Indian (Sikhs) Field Regiment during the violent partition of India. He recalled hearing Jawaharlal Nehru and Mohammed Ali Jinnah address vast crowds and witnessing the birth of two nations. Playing rugby for the British Army, he expected to return home, only to be ordered back to India to finish his National Service. The physical and mental suffering he witnessed during these years called him to medicine, and he later joked that his army uniform helped him get into St Thomas’ Medical School.

Qualifying in 1954, David returned to Wales to work at St David’s Mental Hospital in Carmarthen while his father was dying of silicosis. His early practice involved hypnotic paraldehyde, Drinamyl (later abused as ‘purple hearts’), deep insulin coma therapy and, for the first time, chlorpromazine. After his father’s death he returned to London for further training, working with Roger Tredgold, Desmond Pond and Kenneth Soddy at University College Hospital, the Institute of Psychiatry and Runwell Mental Hospital. At Runwell he witnessed the introduction of imipramine and amitriptyline and the early use of out-patient electroconvulsive therapy. A dynamic clinical environment, and inspirational mentors such as Dr Rolf Strøm-Olsen, fostered his dedication to evidence-based practice.

During his registrar years, David presented the case of a man convinced that his wife was an impostor. His psychodynamic formulation, that the patient’s ‘fallen idol’ had to be split into the loved wife and the hated impostor, won the bronze medal of the Royal Medico-Psychological Association. Fascinated by rare delusions, he researched conditions such as Capgras, De Clérambault’s, folie à deux and Tourette’s. The resulting book, *Some Uncommon Psychiatric Syndromes* (1967), became a bestseller and went through several editions; it has been translated into French, German, Turkish and Japanese. David later observed that Munchausen syndrome by proxy and other disorders that he described ‘became huge’ and that patients and lawyers continued to contact him about them. He also wrote *Healing the Hurt Mind: Christian Faith and Psychiatry* (1983), inspired by a grateful patient who wrote that healing began because David listened, and *I Want a Christian Psychiatrist* (2006), about the interface between faith and mental illness.

Appointed to his first consultant post at Shelton Hospital in Shrewsbury after impressing Sir William Trethowan in a debate about Ganser syndrome, David introduced regular teaching sessions, created therapeutic communities and piloted one of Britain’s earliest care-in-the-community schemes. He met Enoch Powell, who was then advocating the closure of asylums, and joined Jungian psychoanalyst Barbara Robb’s campaign to improve care for older patients. He contributed a chapter to Robb’s book *Sans Everything: A Case to Answer* (1967). The group’s criticisms of neglect and abuse were initially dismissed as exaggerations, but they eventually prompted government inquiries and reforms. In 1971, David became a founding member of the Royal College of Psychiatrists, chairing its north-west division and serving on council and the Court of Electors. He helped develop subspecialty curricula, mentored trainees and donated funds for student fellowships. Colleagues at the new Royal Liverpool University Hospital, where he was head-hunted to create a ‘superb’ department, recall him as a charismatic teacher and visionary leader.

David saw psychiatry as a holistic discipline that must address body, mind and spirit. He insisted that medical students and nurses attend ward rounds so they could see the full range of disorders and learn to listen to patients. He believed that psychiatrists should remain first-class physicians, and often diagnosed physical illnesses missed by others. Asked why he loved psychiatry, he said it was because it deals with the whole person and provides hope for healing. He remained fascinated by consciousness and the brain, lamenting that brain scans had not answered questions about jealousy or monomanias. Even after 50 years in practice, he continued to advocate for keeping psychiatric wards within general hospitals and saw psychiatry as medicine’s most intriguing frontier.

Having seen his last patient on 31 December 2012, David survived a 99% coronary occlusion and continued to lecture at Cardiff University Medical School. He preached regularly, as he had done since the age of 16, and wrote a collection of essays in Welsh exploring the Ten Commandments. In 2022 he published *Enoch’s Walk: Ninety-Five, Not Out*, a memoir that drew together his wartime experiences, clinical adventures and spiritual reflections. Although he never completed that theology degree, he felt greatly blessed and expressed gratitude for a life filled with family, medicine and faith. He is remembered as a compassionate physician, an inspirational teacher, a rigorous scholar and, above all, a champion of human dignity in psychiatry.

David died following a period of declining health. David’s first wife, Joyce, predeceased him in 1992. He later married Anne, a head teacher, who survives him along with his son, Dafydd Enoch K.C., and six grandchildren.

